# The RNA-binding protein NANOS1 controls hippocampal synaptogenesis

**DOI:** 10.1371/journal.pone.0284589

**Published:** 2023-04-14

**Authors:** Darío Maschi, Ana J. Fernández-Alvarez, Graciela Lidia Boccaccio

**Affiliations:** 1 Fundación Instituto Leloir (FIL), Buenos Aires, Argentina; 2 Instituto de Investigaciones Bioquímicas de Buenos Aires (IIBBA) - Consejo Nacional de Investigaciones Científicas y Tecnológicas (CONICET), Buenos Aires, Argentina; 3 Department of Molecular and Cellular Biology and Physiology (FBMyC), Facultad de Ciencias Exactas y Naturales (FCEN), University of Buenos Aires, Buenos Aires, Argentina; John Curtin School of Medical Research, AUSTRALIA

## Abstract

Proteins from the NANOS family are conserved translational repressors with a well-known role in gonad development in both vertebrates and invertebrates. In addition, *Drosophila* Nanos controls neuron maturation and function, and rodent Nanos1 affects cortical neuron differentiation. Here we show that rat *Nanos1* is expressed in hippocampal neurons and that the siRNA-mediated knockdown of *Nanos1* impairs synaptogenesis. We found that both dendritic spine size and number were affected by *Nanos1* KD. Dendritic spines were smaller and more numerous. Moreover, whereas in control neurons most dendritic PSD95 clusters contact pre-synaptic structures, a larger proportion of PSD95 clusters lacked a synapsin counterpart upon *Nanos1* loss-of-function. Finally, *Nanos1* KD impaired the induction of ARC typically triggered by neuron depolarization. These results expand our knowledge on the role of NANOS1 in CNS development and suggest that RNA regulation by NANOS1 governs hippocampal synaptogenesis.

## Introduction

NANOS proteins are evolutionarily conserved RNA regulators mainly known for their significance to gonad development. More incipient is our knowledge on their role in cancer progression or nervous system development. Only one *nanos* gene is present in *Drosophila* and several orthologs are present in vertebrates. Mammalian genomes include three genes termed *NANOS1*, *NANOS2*, and *NANOS3*. The domain organization and interaction motifs of NANOS proteins have been recently reviewed [1]. In both insects and vertebrates, the NANOS N-terminal region can recruit the CCR4-NOT deadenylase complex thus mediating mRNA destabilization [2]. NANOS proteins interact with their target transcripts in multiple ways. A number of NANOS orthologs recruit proteins from the PUMILIO (PUM) and FBF family (PUF), which recognize an 8-nt linear motif present in numerous transcripts (reviewed in [3]). Thus, NANOS can be recruited to mRNAs in an indirect manner. In addition, mammalian NANOS2 and NANOS3 can bind specific RNA consensus motifs and moreover, the interaction of *Drosophila* Nanos with RNA can modulate the binding of Pumilio to the target transcripts [4–6].

Vertebrate and invertebrate NANOS proteins are expressed in primordial and differentiated germ cells, where they are critical for cell survival and pluripotency [1, 7]. The implicated mechanisms are complex and involve multiple target mRNAs and diverse cellular pathways. In *Drosophila* primordial germ cells, Nanos controls the translation of importin-α2 mRNA. Reduced levels of importin-α2 limit the nuclear import of key transcription factors, thereby contributing to the transcriptional repression of somatic genes and preserving pluripotency [8]. Similarly, vertebrate NANOS proteins are required for stem cell maintenance in both male and female gonads, where the three NANOS orthologs have non-redundant functions. Frog, rodent and human NANOS1 repress the translation of mRNAs that induce endoderm differentiation or apoptosis. NANOS2 is male-specific, represses female differentiation and regulates key metabolic pathways, thus promoting the self-renewal of spermatogonial stem cells. In contrast, NANOS3 is important for gonad development in both males and females, where it prevents germ cell apoptosis. NANOS proteins are also implicated in cancer, in connection with their capacity to control cell proliferation, differentiation and apoptosis. Moreover, alterations of NANOS 1, NANOS 2 or NANOS 3 expression levels correlate with tumorigenesis in a complex pattern [1, 4, 9–17].

In addition, NANOS proteins have been implicated in neuronal development and function. *Drosophila* Nanos affects the branching of a particular type of sensory neurons, termed class IV dendritic arborization (C4da) neurons [18, 19]. Either overexpression or loss-of-function of *Drosophila* Nanos reduces dendrite branching. Pumilio is as well implicated and the post-transcriptional repression of the pro-apoptotic gene head involution defective (*hid*), followed by downregulation of caspases is a key pathway in the control of dendrite branching downstream of Nanos/Pumilio [18, 19]. In addition, fly Nanos affects larva neuromuscular junctions (NMJ), with specific effects at both the presynaptic and post-synaptic sides [20]. Loss-of-function of neuronal Nanos correlates with a higher number of NMJ boutons. In the post-synaptic muscle cells, fly Nanos represses the translation of the glutamate receptor IIB (GluRIIB) subunit, thus leading to a larger proportion of receptors containing the GluRIIA subunit. As a result, higher current flows occur upon stimulation of muscular glutamate receptors [20]. Finally, the Nanos/Pumilio repressor complex affects larval motoneurons, where it represses the expression of the sodium channel termed Paralytic, with important physiological consequences [21].

The relevance of NANOS in the vertebrate nervous system is incipiently described, and our current knowledge is largely limited to the role of NANOS1 in the rodent cortex [22]. The three murine orthologs are expressed in embryonic cortical precursors. Whereas the function of NANOS2 and NANOS3 remains unknown, NANOS1 was reported to promote cortical neurogenesis. The loss-of-function of murine NANOS1 increases the number of precursor cells and impairs their transition into neurons. NANOS1 overexpression generates the opposite phenotype [22]. The identity of the relevant mRNAs under NANOS1 control, and whether PUF proteins participate in their regulation remain unknown.

The significance of NANOS1 in other brain areas or developmental stages is poorly described. *Nanos1* mRNA is strongly expressed in the adult mice hippocampus [23]. Here, we confirm the expression of NANOS1 in rat hippocampal neurons in vitro and investigate the consequences of *Nanos1* loss-of-function. We found that the RNAi-mediated knockdown of *Nanos1* affects dendritic spine maturation and impairs neuron stimulation.

## Results

**Fig 1:**
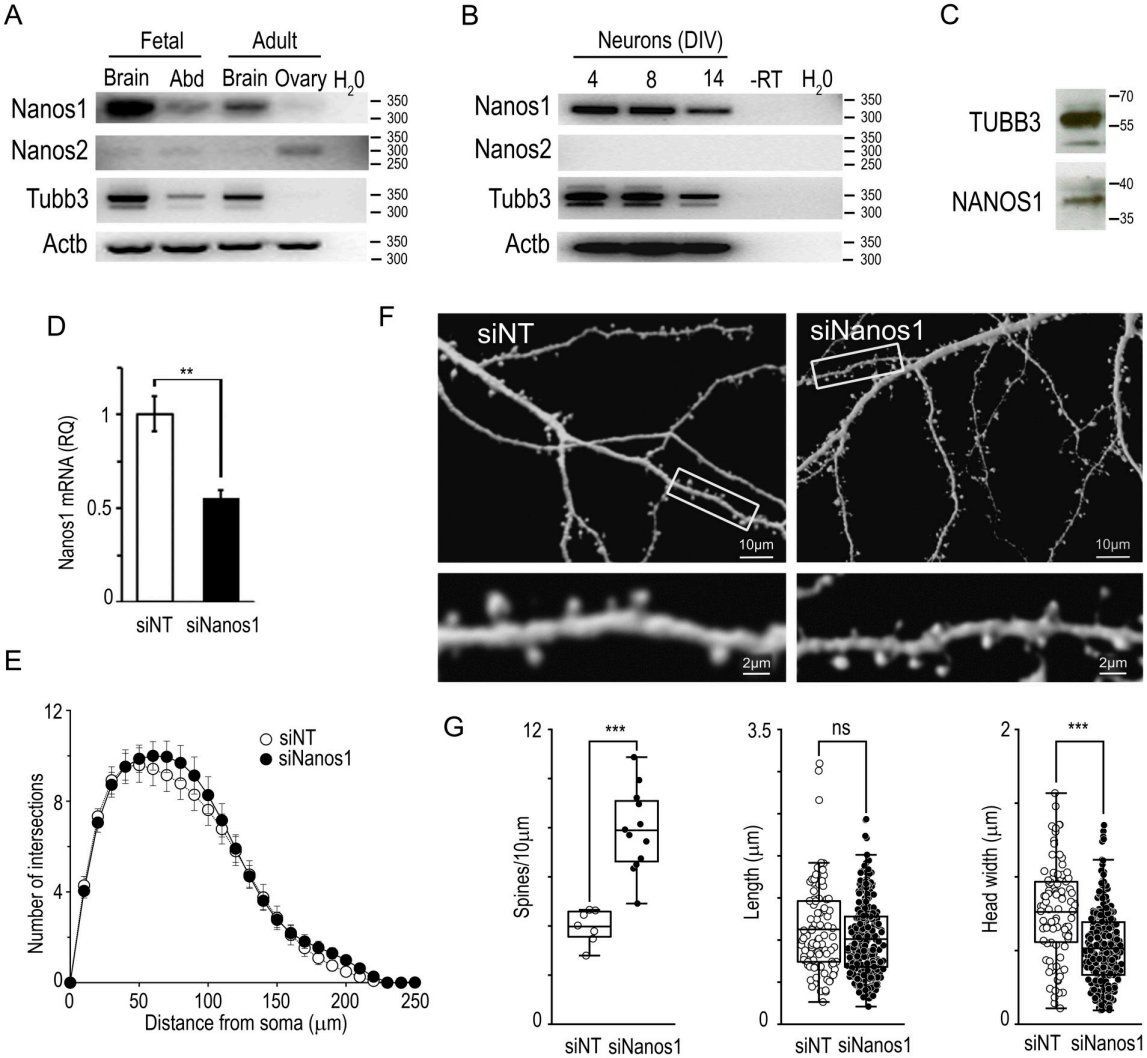
*Nanos1* knockdown affects dendritic spines. (A, B) *Nanos1* expression in rat tissues (A) and cultured hippocampal neurons (B). RT-PCR for *Nanos1*, *Nanos2*, *Tubb3*, or *Actb* was performed, and PCR products were analyzed in agarose gels. A representative experiment out of three is shown. Days in vitro (DIV) are indicated. (C) Protein extracts from 14-DIV cultured neurons were analyzed by western blot for the indicated proteins. (D) Cultured hippocampal neurons were allowed to differentiate for 7 days and then exposed to a pool of four siRNA against *Nanos1* (siNanos1) or a non-targeting siRNA (siNT) as indicated in Materials and Methods. *Nanos1* mRNA levels normalized to *Actb* mRNA levels were analyzed at 14 DIV by quantitative RT-PCR. Error bars, standard deviation from triplicate RT-PCRs, ** p<0.05, Student’s t-test. (E-G) pECFP-N1 was transfected one day after plating and then neurons were treated with the indicated siRNAs as in (D). (E) Dendritic arborization was evaluated by Sholl analysis as indicated in Materials and Methods in 26 siNT and 31 siNanos1-treated cells from six independent cultures. A t-test was performed for each step. (F) Representative dendrite fragments are shown in each case. (G) The number of dendritic spines, their length, and the maximum diameter of the spine heads were determined in three independent experiments (siNT: 91 spines from 7 neurons; siNanos1: 271 spines, 12 neurons), ** p< 0.01; *** p< 0.001, Student’s t-test (Error bars, standard error).

We first investigated the expression of *Nanos1* mRNA by RT-PCR in the rat brain and in cultured hippocampal neurons. As reported before by *in situ* hybridization analysis, we found that *Nanos1* mRNA is strongly expressed in brain, with higher levels at early developmental stages, and very weakly expressed in ovary (Fig 1A) [23]. In developing neurons, *Nanos1* mRNA was detected all along their differentiation *in vitro*, from day 4th to 14th after plating (Fig 1B). In contrast, *Nanos2* mRNA was faintly detected in brain as reported before, and was not detected in developing neurons *in vitro* (Fig 1) [1]. The presence of NANOS1 protein in mature neurons was confirmed by western blot (Fig 1C). To investigate the role of NANOS1 in neuron maturation, we treated neurons that were allowed to differentiate for 7 days *in vitro* (7DIV) with a pool of four siRNAs against *Nanos1*. Quantitative RT-PCR indicated a reduction of *Nanos1* mRNA to about half the normal levels (Fig 1D).

*Drosophila* Nanos was shown to affect the dendritic branching of C4da larval neurons. To evaluate potential effects of mammalian NANOS1 in dendritic branching, we transfected neurons with an ECFP plasmid vector, which facilitates the analysis of dendritic branches and spines, previous to the treatment with either a non-relevant siRNA or the *Nanos1*-specific pool (Fig 1E–1G). Sholl analysis revealed no significant changes in branch complexity (Fig 1E). In contrast, we found important differences in the number and size of dendritic spines (Fig 1F and 1G). Control cells showed 3.9 spines/ 10 μm, a value comparable to those reported previously [24]. Remarkably, the number of spines increased to 7.9 /10 μm upon *Nanos1* KD. In addition, the average size of the spine heads was reduced to about half its normal value (Fig 1G).

**Fig 2:**
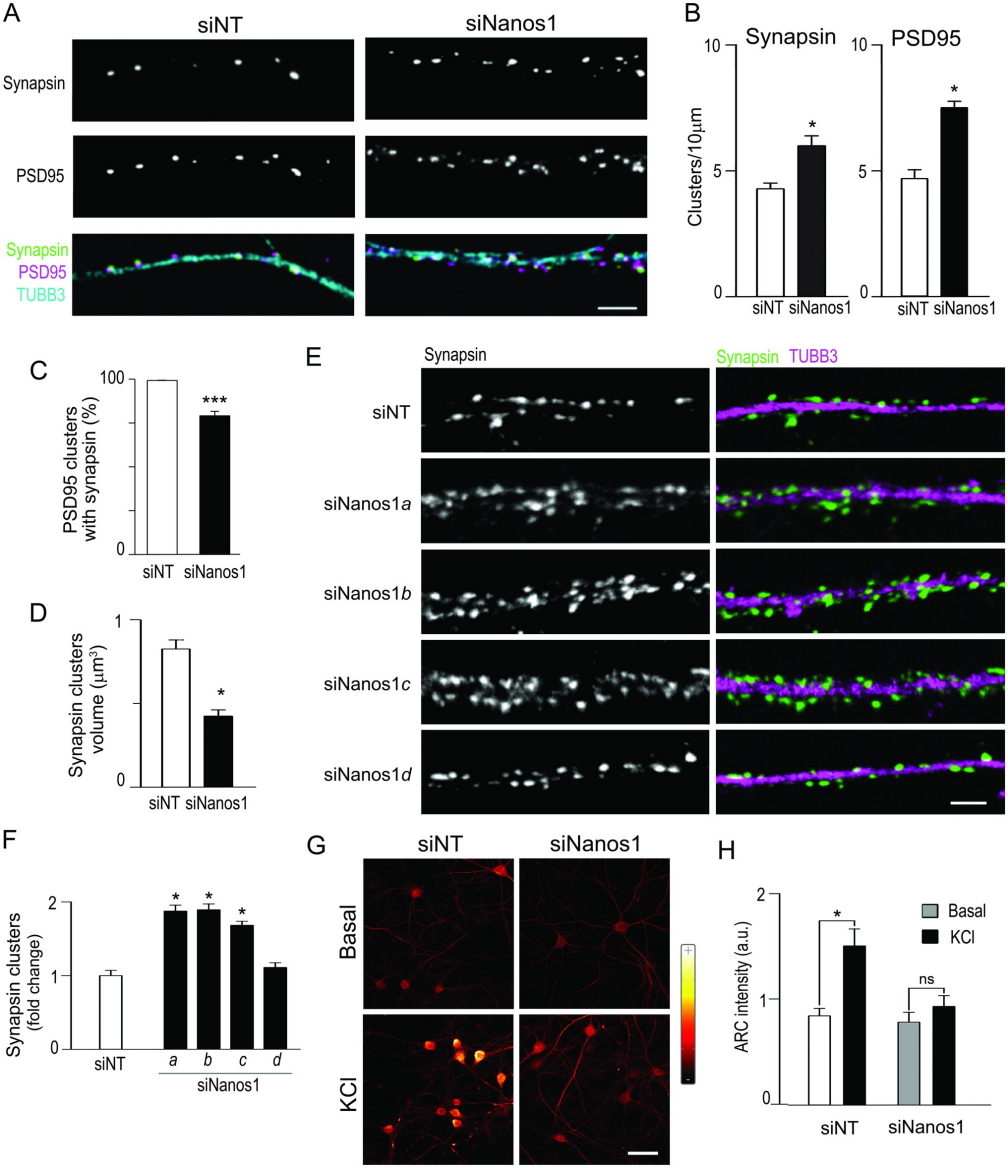
*Nanos1* knockdown affects hippocampal synapses and neuron excitability. (A-D) Neurons were treated with the pool of siRNAs against *Nanos1* (siNanos1) or with the non-targeting siRNA as in Fig 1, and stained for the indicated synapse markers and TUBB3. Three independent experiments were performed. (A) Representative dendrite fragments. Size bar, 5μm. The number of PSD95 or synapsin puncta (B) and the number of PSD95 puncta with a cognate synapsin cluster (C) were analyzed in 10 randomly-selected neurons with a total dendritic length of 4389 μm (siNT) and 5174 μm (siNanos1). (D) The average volume of the synapsin clusters was determined as indicated in Materials and Methods. (E, F) Neurons were treated with each one of the four dsRNA present in the pool used in Fig 2A-2D. (E) Representative dendrite fragments stained for synapsin and TUBB3 are shown. Size bar, 5μm. (F) The number of synapsin puncta was measured in randomly selected dendritic fragments from three independent cultures, with a total dendritic length of 562μm (siNT); 793μm (siNanos1*a*); 642μm (si Nanos1*b*); 590μm (si Nanos1*c*) and 739μm (si Nanos1*d*). * p< 0.05; *** p< 0.001 (ANOVA and Dunnett’s Multiple Comparison test). (G, H) Neurons were treated with the indicated siRNAs and exposed to a repeated depolarizing stimulus as described in Materials and Methods. Immunofluorescence for ARC is depicted in glow scale (G). (H) ARC integrated intensity was determined in the cell body from 100 neurons from duplicate coverslips in 20X micrographs. A representative experiment out of two is depicted, * p< 0.05, Student’s t-test.

This phenotype with smaller and more numerous spines is indicative of defective synaptogenesis. Then, we stained synapsin and post-synaptic density protein 95 (PSD95) to respectively identify pre- and post-synaptic structures, and βIII-tubulin (TUBB3) to identify the dendritic shaft (Fig 2A). We found significant alterations in the size and number of both PSD95 and synapsin puncta. The number of PSD95 clusters increased from 4.7 /10 μm to 7.6 /10 μm. The number of synapsin clusters also increased, although to a lesser extent (Fig 2B). Remarkably, whereas in control neurons almost all PSD95 clusters are adjacent or colocalize with a synapsin cluster, an important fraction (21%) of PSD95 puncta were free of synapsin signal upon *Nanos1* KD (Fig 2C). In addition, we found a 50% reduction in the size of the synapsin patches (Fig 2D).

To control for potential off-target effects, we treated cultured neurons with each one of the four siRNAs against *Nanos1* present in the pool. We found that three of them, siNanos1*a*; siNanos1*b* and siNanos1*c* replicated the phenotype generated by the pool of the four sequences. The number of synapsin clusters significantly increases upon exposure to these individual siRNAs (Fig 2E and 2F). These results strongly support that the effect is elicited by *Nanos1* loss-of-function and not by downregulation of off-target genes. Collectively, the above observations suggest that NANOS1 is required for hippocampal synapse maturation.

Next, we investigated whether neuron activity is compromised upon *Nanos1* KD. We evaluated potential changes in synapse excitability as previously performed [24]. Briefly, we stimulated the neurons with repeated KCl pulses, which triggers a long-term potentiation (LTP)-like response [25], and stained the cells for activity-regulated cytoskeleton-associated protein (ARC), which is an early response gene. As expected, we found that ARC levels doubled in siNT-treated cells (Fig 2G and 2H). In contrast, ARC levels remained unchanged in neurons treated with siNanos1, indicating defective neuron excitability upon *Nanos1* loss-of-function.

## Discussion

Here we show that NANOS1 is expressed during hippocampal neuron development and relevantly, NANOS1 affects synaptogenesis in vitro. *Nanos1* loss-of-function provokes defective spine pruning and impairs synapse maturation. Dendritic spines were smaller and more numerous upon *Nanos1* KD, a larger number of PSD95 puncta was observed, and moreover, a significant proportion of them lacked presynaptic counterparts. Accompanying these alterations, *Nanos1* loss-of-function dampened the response to a depolarizing stimulus.

Which mRNAs under NANOS1 control are causative of defective synaptogenesis remains unknown. Another open question is whether NANOS1 regulation of synapse development involves PUF proteins, which are frequently implicated in post-transcriptional regulation by NANOS and play important roles in both vertebrate and invertebrate neurons. Specifically, *Drosophila* Nanos together with Pumilio forms a repressor complex that regulates C4da neuron dendritic branching. Vertebrate NANOS1 interacts with PUMILIO2 in germ cells, and PUMILIO2 is required for hippocampal neuron growth and synapse development [26–31]. Multiple PUMILIO2 targets are relevant to neuron function and whether these transcripts are dysregulated upon *Nanos1* KD is currently unknown. An interesting candidate for future analysis is the sodium voltage-gated channel alpha subunit 1 (*Scn1a*) mRNA, which contains putative PUMILIO binding sites [32]. Supporting this speculation, the fly sodium channel Paralytic, which is functionally related to mammalian Scn1a, is under the control of Nanos and Pumilio [21, 33]. The neuronal defects generated by mammalian *Nanos1* loss-of-function are compatible with altered excitability as expected to occur upon Scn1a dysregulation. Another putative target is eukaryotic initiation factor 4E (eIF4E) mRNA, which is regulated in mammalian neurons by PUMILIO2 [29]. While future research will inform on the contribution of these and other transcripts, we speculate that multiple mRNAs are likely to be altered upon *Nanos1* loss-of-function [26, 28].

Whether the defective neuronal phenotype is the consequence of presynaptic or post-synaptic alterations is unclear. In *Drosophila* neurons, Nanos function at several locations with different consequences. Whereas neuron morphogenesis requires Nanos activity in dendrites, synaptic function is less affected by loss of Nanos in the dendritic compartment [34]. Vertebrate PUMILIO2 was proposed to prevent the localization of its target transcripts into developing axons [28]. Whether NANOS1 is involved in the spatial restriction of mRNAs mediated by PUMILIO2 remains unknown. In addition, mammalian NANOS1 might act in the control of mRNAs in dendrites, as its partner PUMILIO2 was shown to form dendritic granules [35]. The condensation of granules containing RBPs is a common theme in post-transcriptional regulation, particularly in neuronal dendrites, and whether NANOS1 is present in PUMILO2 granules remains open [36]; reviewed in [37].

Finally, given the conserved role of NANOS proteins in neuron development across evolution, we speculate that their regulation is similarly conserved. In fly embryos and larval neurons, *Drosophila nanos* mRNA is controlled by Smaug, a highly conserved RNA-binding protein. The mammalian Smaug family includes two genes, termed sterile alpha motif domain containing 4A (*SMAUG1/SAMD4A)*, and sterile alpha motif domain containing 4b (*SMAUG2/SAMD4B)*. *Drosophila* and mammalian Smaug proteins bind to a common RNA motif, termed Smaug recognition element (SRE) [36–38], which is present in both fly *nanos* and mammalian *Nanos1* mRNAs [22]. Relevantly, Smaug2 represses *Nanos1* mRNA during cortical neuron differentiation [22]. The expression of both Smaug1 and Smaug2 increases during hippocampal neuron maturation and Smaug1 promotes synapse consolidation [24, 37]. Thus, regulation of *Nanos1* mRNA by Smaug proteins in hippocampal neurons is likely and worthy of future research. In summary, this work describes the relevance of *Nanos1* in hippocampal neuron development, and we further propose that the neuronal Smaug–Nanos-Pumilio axis is conserved in the animal kingdom.

## Materials and methods

### Neuron culture, transfection, and siRNA treatment

All experiments involving animals were conducted according to the protocols approved by the Institutional Animal Care and Use Committee (IACUC) of the Fundación Instituto Leloir. Sprague Dawley pregnant rats were purchased from the Central Animal House at the Facultad de Farmacia y Bioquímica, University of Buenos Aires. Pregnant rats were exposed to CO2 for about one minute until the breathing stopped. Pregnant rats and E18 pups were euthanized by rapid decapitation. Hippocampal neurons were prepared as previously described [24]. In brief, for each preparation, hippocampi were dissected from 5 to 8 rats at embryonic day 18 and digested with trypsin. Cells were seeded on poly-d-lysine (Sigma-Aldrich)-coated glass coverslips. Cultures were maintained in Neurobasal medium (NB; Invitrogen) supplemented with B27 (Invitrogen) and glutamine (complete NB; Invitrogen) at 5% CO2. When required, cells were transfected with pECFP-N1 (Clontech) one day after plating using Lipofectamine 2000 Transfection Reagent (Invitrogen) for 4 h according to the manufacturer’s instructions.

For treatment with siRNAs, hippocampal neurons were allowed to grow for 7 days in vitro, incubated with 100 nM siRNA complexed with Lipofectamine 2000 Transfection Reagent (Invitrogen) for 4 h, and analyzed at 15 d in vitro unless otherwise indicated. Either a pool against rat *Nanos1* (Dharmacon ON-TARGETplusSMARTpool L-086349-01-0010) containing the sequences 5′-UCGCUGAGCUGAACCCAUU-3′ (siNanos1*a*), 5′-GCGCAGCACCAGAGACAAC-3′ (siNanos1*b*), 5′-ACGCUCAUCACCAGGGCUA-3′ (siNanos1*c*), and 5′-GCGACAACGCACACACCAU-3′ (siNanos1*d*) at 25 nM each, or the individual sequences at 100 nM were used. A non-targeting pool (Thermo Fisher Scientific D-001810-10) was used at 100 nM as control.

### Western blot

Whole cell lysates were prepared in RIPA buffer. Western blot was performed by standard procedures using polyvinylidene fluoride membranes (Immobilon-P polyvinylidene difluoride, Millipore). The following antibodies were used: rabbit anti-NANOS1 (Abcam AB65203), 1:100; mouse monoclonal anti TUBB3 (Sigma-Aldrich T8660), 1:400; and HRP-conjugated secondary antibodies (Jackson 715-035-150 and 111-035-144), 1:10,000. ECL Prime (GE Healthcare) was used.

### Neuron stimulation

Neuron stimulation by repeated depolarization was induced as described (Wu et al., 2001). In brief, mature hippocampal neurons were treated with 1 μM Tetrodotoxin (TTX) for one day to block spontaneous activity. Then, repeated depolarization stimuli with 90 mM KCl in isotonic Tyrode’s solution for 3 min were applied four times at 10 min intervals. TTX was applied during the resting intervals and after the stimulation phase. Neurons were analyzed one hour after stimulation.

### Immunofluorescence and image analysis

Immunofluorescence of cultured neurons was performed after fixation, permeabilization, and blocking as previously described [24, 39]. Primary antibodies were diluted as follows: monoclonal IgG2a anti-PSD95 (Millipore 05–494), 1:100; IgG2b anti-TUBB3 (Sigma-Aldrich T8660), 1:500; IgG1 anti-synapsin (Synaptic Systems 106001), 1:100; polyclonal anti-ARC (Synaptic Systems 156003), 1:500; anti-GFP (Invitrogen A6455), 1:100. Secondary antibodies coupled to Alexa Fluor 488 used at 1:1,000 were obtained from Invitrogen. Secondary antibodies coupled to Cy2, Cy3, or Cy5, used at 1:300–1:500, were from Jackson ImmunoResearch Laboratories.

Images were acquired with an LSM510 Meta confocal microscope (Carl Zeiss), using Plan-Apochromat 63X/1.4 NA oil objective lenses and LSM software (Carl Zeiss) unless otherwise indicated. Pixel intensity was always lower than 250, with 255 being the level of saturation. Equipment adjustment was assessed by using 1 μm FocalCheck fluorescent microspheres (Invitrogen). No filters or gamma adjustments were used for the analysis of the object’s size, number, or intensity, which were analyzed with the ImageJ software (https://imagej.net/ij/index.html).

Sholl analysis: For each neuron, we retrieved the metrics calculated by the Sholl Analysis Fiji plug-in (https://imagej.net/plugins/sholl-analysis) as described [40]. We quantified arborization in 10μm increments from 0 to 300 μm from the soma. Dendritic spine parameters were determined by combining Scientific Volume Imaging’s Huygens software for image deconvolution and Imaris software for 3D rendering and quantification.

For the analysis of synapsin clusters, 10 confocal planes with an axial separation of 0.41μm, which include the whole volume of the synapsin signal were used. The X and Y pixel size was 0.09μm. Image deconvolution was performed using the Huygens software (SVI—Scientific Volume Imaging) to improve lateral and axial resolution. Then, a 3D reconstruction was rendered and the volume of the synapsin cluster was determined.

### RT-PCR and qRT-PCR

For both RT-PCR and qRT-PCR, total RNA was isolated using TRIzol reagent (Invitrogen). First-strand cDNA was synthesized from 1 μg of total RNA using a random hexamer and expand reverse transcriptase (MMLV, Promega). The cDNA was used as a template for PCR or quantitative PCR. Quantitative PCR was performed using Syber Green reagent (Roche). Primers for endpoint PCR were as follows: *Nanos1*, Fw 5'-GGTATCAAGCCAGGATTGCT-3', Rv 5'-CTGGGGTTATAGGCGCATGAC-3'; *Nanos2*, Fw 5'-ACATAAGTGTCATGGACCTGC-3', Rv 5'-CATACGTAATGCCTCAGGATGG-3'; *Tubb3* Fw 5'-CCTGGAACCATGGACAGCGTTCG3', Rv 5'-CGTTGTAGGGCTCTACCACGGTG-3'; *Actb*: Fw 5'-ACTATCGGCAATGAGCGGTTCC3' Rv 5'-GGACTCATCGTACTCCTGCT3'. Primers for real time PCR were as follows: *Nanos1*, Fw 5'-CTCTTGGTTTTATGGAAGCCGCA-3', Rv 5'-GCACTTAAAATAGGCTGACGT-3'; *Actb*, Fw 5'-TGTCACCAACTGGGACGATA3', Rv 5'-GGGGTGTTGAAGGTCTCAAA3'.

### Statistics

The number of PSD95 or synapsin puncta and dendritic spine parameters were determined with the help of ImageJ. The number of PSD95 and synapsin puncta were statistically analyzed with ANOVA and Dunnett’s Multiple Comparison test. For dendritic spine morphology, the indicated number of images were analyzed by Student’s t-test. ARC intensity was determined in cell bodies and statistically analyzed by Student’s t-test. Instat software (GraphPad Software, Inc.) was used.

## Supporting information

S1 Raw images (PDF)

## References

[1] Ilaslan E, Sajek MP, Jaruzelska J, Kusz-Zamelczyk K. Emerging Roles of NANOS RNA-Binding Proteins in Cancer. Int J Mol Sci. 2022;23(16). doi: 10.3390/ijms23169408 36012673 PMC9409212

[2] Raisch T, Bhandari D, Sabath K, Helms S, Valkov E, Weichenrieder O, et al. Distinct modes of recruitment of the CCR4-NOT complex by Drosophila and vertebrate Nanos. EMBO J. 2016;35(9):974–90. doi: 10.15252/embj.201593634 26968986 PMC5207322

[3] De Keuckelaere E, Hulpiau P, Saeys Y, Berx G, van Roy F. Nanos genes and their role in development and beyond. Cell Mol Life Sci. 2018;75(11):1929–46. doi: 10.1007/s00018-018-2766-3 29397397 PMC11105394

[4] Codino A, Turowski T, van de Lagemaat LN, Ivanova I, Tavosanis A, Much C, et al. NANOS2 is a sequence-specific mRNA-binding protein that promotes transcript degradation in spermatogonial stem cells. iScience. 2021;24(7):102762. doi: 10.1016/j.isci.2021.102762 34278268 PMC8271163

[5] Sajek M, Janecki DM, Smialek MJ, Ginter-Matuszewska B, Spik A, Oczkowski S, et al. PUM1 and PUM2 exhibit different modes of regulation for SIAH1 that involve cooperativity with NANOS paralogues. Cell Mol Life Sci. 2019;76(1):147–61. doi: 10.1007/s00018-018-2926-5 30269240 PMC11105465

[6] Weidmann CA, Qiu C, Arvola RM, Lou TF, Killingsworth J, Campbell ZT, et al. Drosophila Nanos acts as a molecular clamp that modulates the RNA-binding and repression activities of Pumilio. Elife. 2016;5. doi: 10.7554/eLife.17096 27482653 PMC4995099

[7] Mercer M, Jang S, Ni C, Buszczak M. The Dynamic Regulation of mRNA Translation and Ribosome Biogenesis During Germ Cell Development and Reproductive Aging. Front Cell Dev Biol. 2021;9:710186. doi: 10.3389/fcell.2021.710186 34805139 PMC8595405

[8] Asaoka M, Hanyu-Nakamura K, Nakamura A, Kobayashi S. Maternal Nanos inhibits Importin-alpha2/Pendulin-dependent nuclear import to prevent somatic gene expression in the Drosophila germline. PLoS Genet. 2019;15(5):e1008090. 31091233 10.1371/journal.pgen.1008090 PMC6519790

[9] Ilaslan E, Smialek MJ, Sajek MP, Kotecki M, Ginter-Matuszewska B, Krainski P, et al. Human NANOS1 Represses Apoptosis by Downregulating Pro-Apoptotic Genes in the Male Germ Cell Line. Int J Mol Sci. 2020;21(8). 32344590 10.3390/ijms21083009 PMC7215683

[10] Kodama M, Yoshida M, Endo M, Kobayashi T, Oike A, Yasumasu S, et al. Nanos3 of the frog Rana rugosa: Molecular cloning and characterization. Dev Growth Differ. 2018;60(2):112–20. doi: 10.1111/dgd.12421 29405266

[11] Lai F, King ML. Repressive translational control in germ cells. Mol Reprod Dev. 2013;80(8):665–76. doi: 10.1002/mrd.22161 23408501

[12] Lai F, Singh A, King ML. Xenopus Nanos1 is required to prevent endoderm gene expression and apoptosis in primordial germ cells. Development. 2012;139(8):1476–86. doi: 10.1242/dev.079608 22399685 PMC3308181

[13] Sada A, Suzuki A, Suzuki H, Saga Y. The RNA-binding protein NANOS2 is required to maintain murine spermatogonial stem cells. Science. 2009;325(5946):1394–8. doi: 10.1126/science.1172645 19745153

[14] Saga Y. Sexual development of mouse germ cells: Nanos2 promotes the male germ cell fate by suppressing the female pathway. Dev Growth Differ. 2008;50 Suppl 1:S141–7. doi: 10.1111/j.1440-169X.2008.01009.x 18430166

[15] Suzuki H, Sada A, Yoshida S, Saga Y. The heterogeneity of spermatogonia is revealed by their topology and expression of marker proteins including the germ cell-specific proteins Nanos2 and Nanos3. Dev Biol. 2009;336(2):222–31. doi: 10.1016/j.ydbio.2009.10.002 19818747

[16] Wright D, Kiso M, Saga Y. Genetic and structural analysis of the in vivo functional redundancy between murine NANOS2 and NANOS3. Development. 2021;148(1). doi: 10.1242/dev.191916 33199444

[17] Ilaslan E, Kwiatkowska K, Smialek MJ, Sajek MP, Lemanska Z, Alla M, et al. Distinct Roles of NANOS1 and NANOS3 in the Cell Cycle and NANOS3-PUM1-FOXM1 Axis to Control G2/M Phase in a Human Primordial Germ Cell Model. Int J Mol Sci. 2022;23(12). 10.3390/ijms23126592 PMC9223905 35743036

[18] Ye B, Petritsch C, Clark IE, Gavis ER, Jan LY, Jan YN. Nanos and Pumilio are essential for dendrite morphogenesis in Drosophila peripheral neurons. Curr Biol. 2004;14(4):314–21. doi: 10.1016/j.cub.2004.01.052 14972682

[19] Bhogal B, Plaza-Jennings A, Gavis ER. Nanos-mediated repression of hid protects larval sensory neurons after a global switch in sensitivity to apoptotic signals. Development. 2016;143(12):2147–59. doi: 10.1242/dev.132415 27256879 PMC4920170

[20] Menon KP, Andrews S, Murthy M, Gavis ER, Zinn K. The translational repressors Nanos and Pumilio have divergent effects on presynaptic terminal growth and postsynaptic glutamate receptor subunit composition. J Neurosci. 2009;29(17):5558–72. doi: 10.1523/JNEUROSCI.0520-09.2009 19403823 PMC2750846

[21] Muraro NI, Weston AJ, Gerber AP, Luschnig S, Moffat KG, Baines RA. Pumilio binds para mRNA and requires Nanos and Brat to regulate sodium current in Drosophila motoneurons. J Neurosci. 2008;28(9):2099–109. doi: 10.1523/JNEUROSCI.5092-07.2008 18305244 PMC2323674

[22] Amadei G, Zander MA, Yang G, Dumelie JG, Vessey JP, Lipshitz HD, et al. A Smaug2-Based Translational Repression Complex Determines the Balance between Precursor Maintenance versus Differentiation during Mammalian Neurogenesis. J Neurosci. 2015;35(47):15666–81. doi: 10.1523/JNEUROSCI.2172-15.2015 26609159 PMC6705466

[23] Haraguchi S, Tsuda M, Kitajima S, Sasaoka Y, Nomura-Kitabayashid A, Kurokawa K, et al. nanos1: a mouse nanos gene expressed in the central nervous system is dispensable for normal development. Mech Dev. 2003;120(6):721–31. doi: 10.1016/s0925-4773(03)00043-1 12834871

[24] Baez MV, Luchelli L, Maschi D, Habif M, Pascual M, Thomas MG, et al. Smaug1 mRNA-silencing foci respond to NMDA and modulate synapse formation. J Cell Biol. 2011;195(7):1141–57. doi: 10.1083/jcb.201108159 22201125 PMC3246892

[25] Wu GY, Deisseroth K, Tsien RW. Spaced stimuli stabilize MAPK pathway activation and its effects on dendritic morphology. Nat Neurosci. 2001;4(2):151–8. doi: 10.1038/83976 11175875

[26] Dong H, Zhu M, Meng L, Ding Y, Yang D, Zhang S, et al. Pumilio2 regulates synaptic plasticity via translational repression of synaptic receptors in mice. Oncotarget. 2018;9(63):32134–48. doi: 10.18632/oncotarget.24345 30181804 PMC6114944

[27] Follwaczny P, Schieweck R, Riedemann T, Demleitner A, Straub T, Klemm AH, et al. Pumilio2-deficient mice show a predisposition for epilepsy. Dis Model Mech. 2017;10(11):1333–42. doi: 10.1242/dmm.029678 29046322 PMC5719250

[28] Martinez JC, Randolph LK, Iascone DM, Pernice HF, Polleux F, Hengst U. Pum2 Shapes the Transcriptome in Developing Axons through Retention of Target mRNAs in the Cell Body. Neuron. 2019;104(5):931–46.e5. doi: 10.1016/j.neuron.2019.08.035 31606248 PMC6895424

[29] Schieweck R, Schoneweiss EC, Harner M, Rieger D, Illig C, Sacca B, et al. Pumilio2 Promotes Growth of Mature Neurons. Int J Mol Sci. 2021;22(16). doi: 10.3390/ijms22168998 34445704 PMC8396670

[30] Vessey JP, Schoderboeck L, Gingl E, Luzi E, Riefler J, Di Leva F, et al. Mammalian Pumilio 2 regulates dendrite morphogenesis and synaptic function. Proc Natl Acad Sci U S A. 2010;107(7):3222–7. doi: 10.1073/pnas.0907128107 20133610 PMC2840302

[31] Fiore R, Rajman M, Schwale C, Bicker S, Antoniou A, Bruehl C, et al. MiR-134-dependent regulation of Pumilio-2 is necessary for homeostatic synaptic depression. EMBO J. 2014;33(19):2231–46. doi: 10.15252/embj.201487921 25097251 PMC4282509

[32] Thomas MG, Pascual ML, Maschi D, Luchelli L, Boccaccio GL. Synaptic control of local translation: the plot thickens with new characters. Cell Mol Life Sci. 2014;71(12):2219–39. doi: 10.1007/s00018-013-1506-y 24212248 PMC11113725

[33] Driscoll HE, Muraro NI, He M, Baines RA. Pumilio-2 regulates translation of Nav1.6 to mediate homeostasis of membrane excitability. J Neurosci. 2013;33(23):9644–54. doi: 10.1523/JNEUROSCI.0921-13.2013 23739961 PMC3678506

[34] Xu X, Brechbiel JL, Gavis ER. Dynein-dependent transport of nanos RNA in Drosophila sensory neurons requires Rumpelstiltskin and the germ plasm organizer Oskar. J Neurosci. 2013;33(37):14791–800. doi: 10.1523/JNEUROSCI.5864-12.2013 24027279 PMC3771026

[35] Vessey JP, Vaccani A, Xie Y, Dahm R, Karra D, Kiebler MA, et al. Dendritic localization of the translational repressor Pumilio 2 and its contribution to dendritic stress granules. J Neurosci. 2006;26(24):6496–508. doi: 10.1523/JNEUROSCI.0649-06.2006 16775137 PMC6674044

[36] Fernandez-Alvarez AJ, Gabriela Thomas M, Pascual ML, Habif M, Pimentel J, Corbat AA, et al. Smaug1 membrane-less organelles respond to AMPK and mTOR and affect mitochondrial function. J Cell Sci. 2022;135(1). doi: 10.1242/jcs.253591 34859817

[37] Fernandez-Alvarez AJ, Pascual ML, Boccaccio GL, Thomas MG. Smaug variants in neural and non-neuronal cells. Commun Integr Biol. 2016;9(2):e1139252. doi: 10.1080/19420889.2016.1139252 27195061 PMC4857778

[38] Baez MV, Boccaccio GL. Mammalian Smaug is a translational repressor that forms cytoplasmic foci similar to stress granules. J Biol Chem. 2005;280(52):43131–40. doi: 10.1074/jbc.M508374200 16221671

[39] Luchelli L, Thomas MG, Boccaccio GL. Synaptic control of mRNA translation by reversible assembly of XRN1 bodies. J Cell Sci. 2015;128(8):1542–54. doi: 10.1242/jcs.163295 25736288

[40] Ferreira TA, Blackman AV, Oyrer J, Jayabal S, Chung AJ, Watt AJ, et al. Neuronal morphometry directly from bitmap images. Nat Methods. 2014;11(10):982–4. doi: 10.1038/nmeth.3125 25264773 PMC5271921

