# Supplementary figures and images for: The RNA-binding protein NANOS1 controls hippocampal synaptogenesis
Source: PLoS One. 2023 Apr 14;18(4):e0284589. doi: 10.1371/journal.pone.0284589 (PMC10104283; doi:10.1371/journal.pone.0284589)

Figure 1A

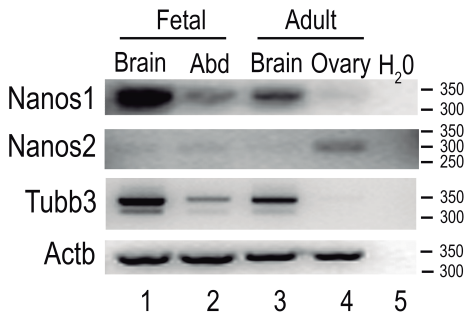

Figure 1B

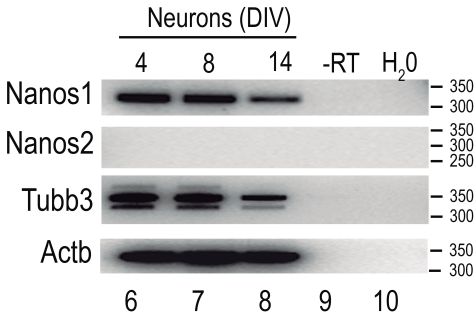

Original images

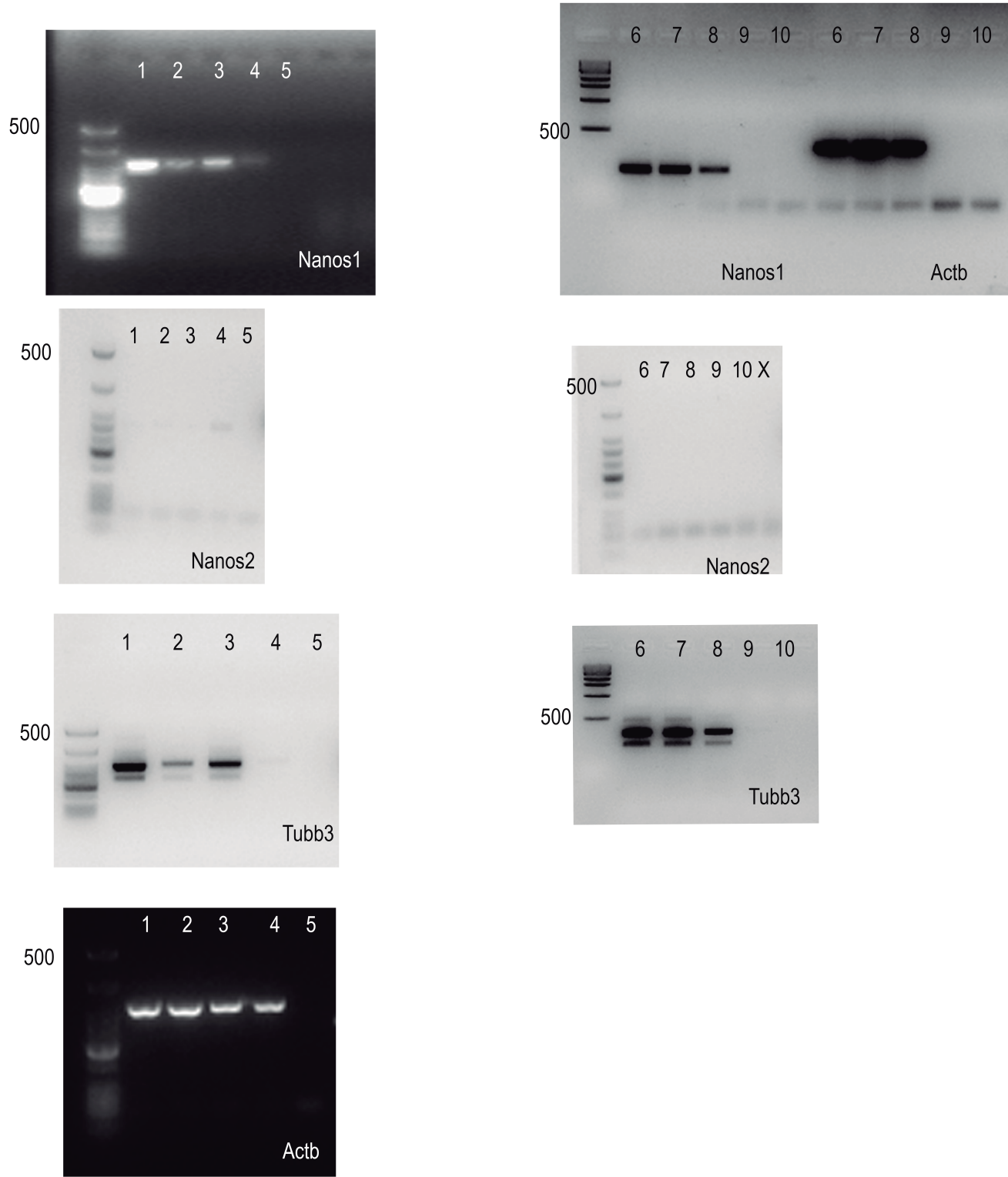

Figure 1C

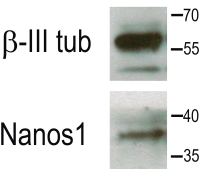

Original images

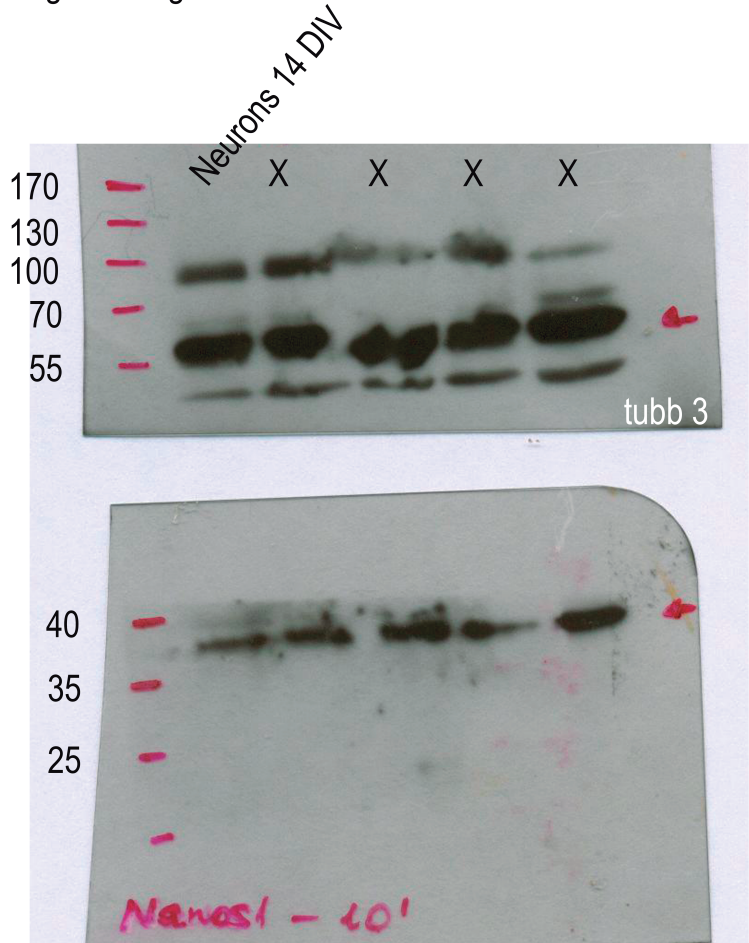

Supplement: S1 Raw images — (PDF) [file pone.0284589.s001.pdf]
